# ZFLNC: a comprehensive and well-annotated database for zebrafish lncRNA

**DOI:** 10.1093/database/bay114

**Published:** 2018-10-18

**Authors:** Xiang Hu, Wen Chen, Jing Li, Shulan Huang, Xuling Xu, Xuan Zhang, Shuanglin Xiang, Changning Liu

**Affiliations:** 1 CAS Key Laboratory of Tropical Plant Resources and Sustainable Use, Xishuangbanna Tropical Botanical Garden, Chinese Academy of Sciences, Kunming, China; 2 State Key Laboratory of Developmental Biology of Freshwater Fish, School of Life Sciences, Hunan Normal University, Changsha, China

## Abstract

There is emerging evidence showing that lncRNAs can be involved in various critical biological processes. Zebrafish is a fully developed model system being used in a variety of basic research and biomedical studies. Hence, it is an ideal model organism to study the functions and mechanisms of lncRNAs. Here, we constructed ZFLNC—a comprehensive database of zebrafish lncRNA that is dedicated to providing a zebrafish-based platform for deep exploration of zebrafish lncRNAs and their mammalian counterparts to the relevant academic communities. The main data resources of lncRNAs in this database come from the NCBI, Ensembl, NONCODE, zflncRNApedia and literature. We also obtained lncRNAs as a supplement by analysing RNA-Seq datasets from SRA database. With these IncRNAs, we further carried out expression profiling, co-expression network prediction, Gene Ontology (GO)/Kyoto Encyclopedia of Genes and Genomes (KEGG)/Online Mendelian Inheritance in Man (OMIM) annotation and conservation analysis. As far as we know, ZFLNC is the most comprehensive and well-annotated database for zebrafish lncRNA.

## Introduction

Long non-coding RNAs (lncRNAs), which were once regarded as ‘junk sequences’, are defined as transcripts longer than 200 nucleotides that have no/low potential for protein coding (1–3). There has been an increasing evidence that lncRNAs are involved in various critical biological processes, such as cancer progression (4, 5), cell differentiation and development (6–8), innate immunity (9, 10), etc. With an enormous range of applications of next generation sequencing, a large number of lncRNAs have been found in human (11, 12), mouse (13, 14), zebrafish (15–17), etc. At the same time, a large number of lncRNA-related databases have been created and are committed to large-scale collection and annotation of lncRNAs for various species. For example, NONCODE contained more than 350 000 lncRNA genes across 17 species including human and zebrafish (18–20). lncRNAdb manually collected and annotated about 300 functional lncRNAs that have been experimentally characterized with a biological function (21). By systematically integrating and sorting the resources of lncRNAs among various species, it could provide the reference information when further probing lncRNAs’ functions and molecular mechanisms, even when performing cross-species functional verification in model organisms.

Zebrafish has a relatively long history as a powerful model for studying vertebrate biology and human diseases, because of its clear development pattern and genetic background. The functions and mechanisms of many coding genes in development and diseases have been elucidated in the zebrafish model (22). Thus, it is worth expecting that zebrafish could continually contribute to revealing lncRNA’s function and mechanism as a competent model system. However, studies of using the zebrafish as a model to probe lncRNA’s function are very limited. Until now, only a small amount of large-scale gene discovery studies and very few gene function experiments for zebrafish lncRNAs have been reported (15–17, 23–25), which is extremely disproportionate to the importance of zebrafish as an extensively studied model organism. We assumed that this problem is partly because of the lack of systematic survey and the insufficient annotation for zebrafish lncRNAs.

Before us, zebrafish lncRNAs were dispersed in different databases and the quantity and annotation information were far less than that of human or mouse lncRNAs. NCBI (26) and Ensembl (27) are two comprehensive databases for all genes, which collect both coding and noncoding gene sequences; NONCODE (18–20) is a multi-species lncRNA database and mainly focused on mammalian lncRNAs; zflncRNApedia (28) is a specialized zebrafish lncRNA database with manually curated resource for lncRNAs in zebrafish, but its information content is far from sufficiency and comprehensiveness. In this study, we constructed ZFLNC (http://zflnc.org)—a comprehensive database of zebrafish lncRNAs coupled with conservation analysis for potential orthologs in human or mouse. In ZFLNC, we have collected the most complete dataset of zebrafish lncRNAs, by comprehensively integrating various data resources from literature, databases and public RNA-seq datasets. ZFLNC also provided the most comprehensive annotations of zebrafish lncRNAs, which include the expression profile, co-expression network, GO/KEGG/OMIM annotation and conservation analysis. We believe ZFLNC will provide a valuable and unique resource and an important platform for further molecular and bioinformatics research of zebrafish lncRNAs as well as their mammalian counterparts.

### Aims of database

Zebrafish is a powerful model system for studying human diseases. Recently, the fact that some lncRNAs are conserved between zebrafish and human and shared with similar functions, has attracted widespread attention. In the case of tuna gene in zebrafish, its knockdown in zebrafish caused impaired locomotor function, and its ortholog (TUNA) expression in human brains of Huntington’s disease patients was significantly associated with disease grade (23). However, there are only a limited number of researches conducted on zebrafish lncRNAs and it is still a big challenge to find conserved functional lncRNA candidate for further experimental investigation.

In order to set up a zebrafish-based platform for deep exploration of the functions and mechanisms of zebrafish lncRNAs and their mammalian counterparts, we constructed ZFLNC, which is a comprehensive database of zebrafish lncRNA with three main goals: (i) collecting the most complete dataset of zebrafish lncRNAs, with the most comprehensive annotations, (ii) Using a variety of conservation analysis methods to study the potential lncRNA orthology and (iii) providing a user-friendly website with useful web-based tools for the functional interrogation of conserved lncRNAs.

### Implementation of the database

#### 

**Figure 1:**
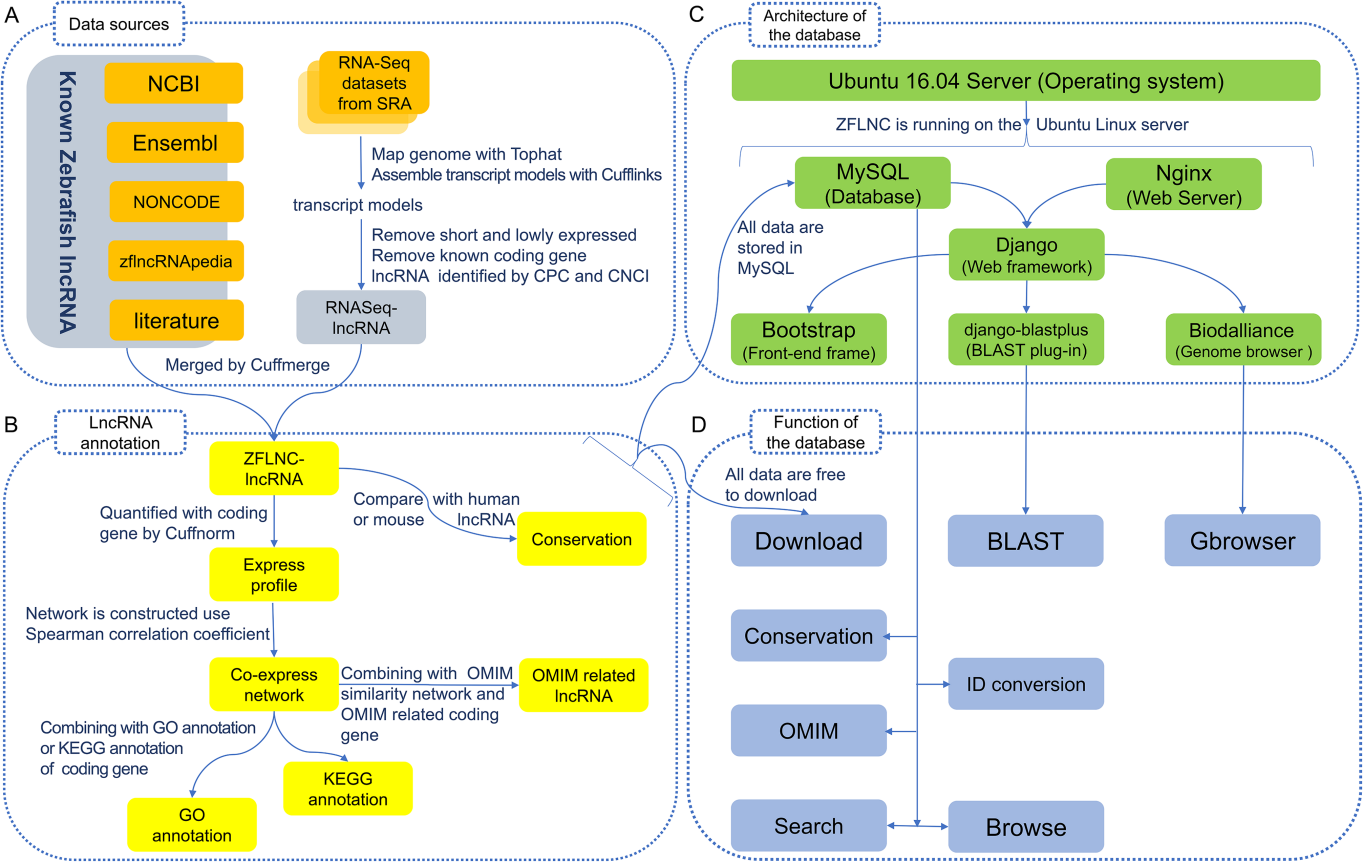
Implementation of the database. **(A)** Data sources. **(B)** LncRNA annotation. **(C)** Architecture of the database. **(D)** Function of the database.

***Data sources and integration.*** The principal data resources of lncRNAs in this database come from NCBI (26), Ensembl (27), NONCODE v4 (20), zflncRNApedia (28) and literature (15, 16). We also obtained lncRNAs as a supplement by analysing RNA-Seq datasets from SRA database (Figure 1A). In total, RNA-seq datasets covered 499 runs in 56 studies from NCBI SRA database (please read the online help manuals for more details). RNA-Seq reads were mapped to Zv9 genome using Tophat2 (29), then performed transcript reconstruction by Cufflinks suite (30), and the derived lncRNAs were identified by CPC (31) and CNCI (32) as described previously (33). We obtained 7394 zebrafish lncRNA genes (13 166 transcripts) from RNA-Seq data and then integrated them with those from Ensembl, NONCODE, NCBI, zflncRNApedia and literature. Compared with the existing database, our ZFLNC includes more lncRNA genes and transcripts (Table 1). In the current version, ZFLNC contains 13 604 lncRNA genes and 21 128 lncRNA transcripts (Table 2).

**Table 1:** Comparison between ZFLNC and related databases

Database	Genes	Transcripts	Expression	Function annotation	Conservation
NCBI	3208	4869	×	×	×
Ensembl	2839	4133	×	×	×
NONCODE	NA	8360	×	×	×
zflncRNApedia	NA	2181	√	×	×
ZFLNC	13 604	21 128	√	√	√

The above information is based on NCBI *Danio rerio* Annotation Release 104, Ensembl Zebrafish release 79, NONCODEv4 and zflncRNApedia. The zebrafish lncRNAs in NONCODEv4 and zflncRNApedia are transcripts level.

#### 

***LncRNA annotation.*** For most biologists, it is difficult to apply a database that only has sequence information with little annotation information. To better optimize user accessibility and availability, ZFLNC is equipped with multiple information of zebrafish lncRNAs, including expression profile, co-expression network, GO/KEGG/OMIM annotations and conservation of lncRNAs, for enriching functional annotations (Figure 1B). So far, ZFLNC has provided the most comprehensive annotations of zebrafish lncRNAs as compared with the existing databases (Table 1).

We quantified the zebrafish coding genes and lncRNAs in different tissues and conditions, so as to construct a co-expression profiling between coding and lncRNA genes. Combining with the annotation of coding genes, we performed GO and KEGG annotation for lncRNAs using network-based prediction methods (33). The GO annotation of zebrafish lncRNA was predicted using the goatools (version 0.6.4) (34), which determines the GO annotation of one gene according to the GO annotations of its co-expression coding genes (*P*-value < 0.05). The KEGG annotation of zebrafish lncRNA was predicted using the in-house Python script. The KEGG annotation of one gene was determined by the enrichment of KEGG annotations according to its co-expression coding genes using hypergeometric distribution (*P*-value < 0.05). In this way, we achieved a set of GO (7345 genes) and KEGG (7055 genes) annotation for zebrafish lncRNAs (Table 2).

We use the random walk with restart on heterogeneous network algorithm (35) to analyse the relationship between lncRNA and OMIM in MATLAB. The upper subnetwork is coding-lncRNA gene co-expression network and the lower network is OMIM similarity network. OMIM similarity matrix is from Disimweb (36) and gene–OMIM relationship is from InterMine (37). With this approach, 291 lncRNA genes are predicted to be OMIM related (Table 2).

To examine the sequence conservation of lncRNAs, we used the phastCons scores calculated from the UCSC 8-way vertebrate genome alignment. We further used three methods [that are direct BLASTN, collinearity with conserved coding gene and overlap with multispecies ultra-conserved noncoding elements (UCNE)] to find the counterparts of zebrafish lncRNAs in human or mouse (38). In direct comparison of zebrafish lncRNA and human/mouse lncRNA with BLASTN, bidirectional best hits using a relatively nonstringent threshold (E-value < = 10^−5^) were considered as orthologs. In collinearity method, we compared the coding genes of zebrafish with those of human or mouse using BLASTP as anchor points. We assumed that those lncRNAs with more than five anchor points in the 20 k upstream/downstream region are orthologs. In UCNE method, if two lncRNAs from different species overlap with at least one UCNE, as another anchor point, they are considered as orthologs. Finally, we obtained 2156 zebrafish lncRNA genes that have the counterpart in human or mouse (Table 2).

**Table 2:** The statistics of ZFLNC

lncRNA	Number
All genes	13 604
All transcripts	21 128
Genes with GO annotation	7345
Genes with KEGG annotation	7055
Genes with putative OMIM	291
Putative conserved genes	2155

#### 

***Function of the database.*** For biologists to better access zebrafish lncRNA information, we established a user-friendly website. In addition to the basic browsing, searching and download services, we offer online BLAST service, Genome Browse Server, ID conversion and ‘Advanced functional lncRNA filtering’, and each section has enough help information, such as data sources, data processing and database usage (Figure 1D).

In ‘Browse’ module, you can browse all lncRNA genes or transcripts. LncRNA is sorted according to the richness of its annotation. In particular, you can also browse lncRNAs with conservation or OMIM annotation directly in ‘Conservation’ and ‘OMIM’ modules. ‘Search’ module provides a simple and fast search based on lncRNA ID and also an ‘Advanced functional lncRNA filtering’ function to help you find interested lncRNAs in according to its expression profile in tissue, the co-expressed coding gene and the annotated biological function. In ‘GBrowser’ module, you can view lncRNA-related genomic annotation, such as mRNA, conserved non-genic elements, genome variation and miRNA. ‘BLAST’ module can query ZFLNC based on sequence similarity. ‘ID Conversion’ module can convert the ID of lncRNA in other databases and ZFLNC. All data of ZFLNC are free to download.

#### 

***Architecture of the database.*** ZFLNC (http://www.zflc.org) is an open-access database implemented by some free and open-source software (Figure 1C). The website was developed using Python (https://www.python.org/) based on Django web framework (http://www.djangoproject.com/). The website is running on Ubuntu Linux server (https://www.ubuntu.com/), while Nginx web server (https://www.nginx.com/) as web server and SQLite (https://www.sqlite.org/) as database server. The web front-end was developed using Bootstrap framework (http://getbootstrap.com/). The GBrowser function was developed using Biodalliance (39) (http://www.biodalliance.org/). The BLAST function was developed using django-blastplus (https://pypi.python.org/pypi/django-blastplus/).

### Usage of the database

#### 

**Figure 2:**
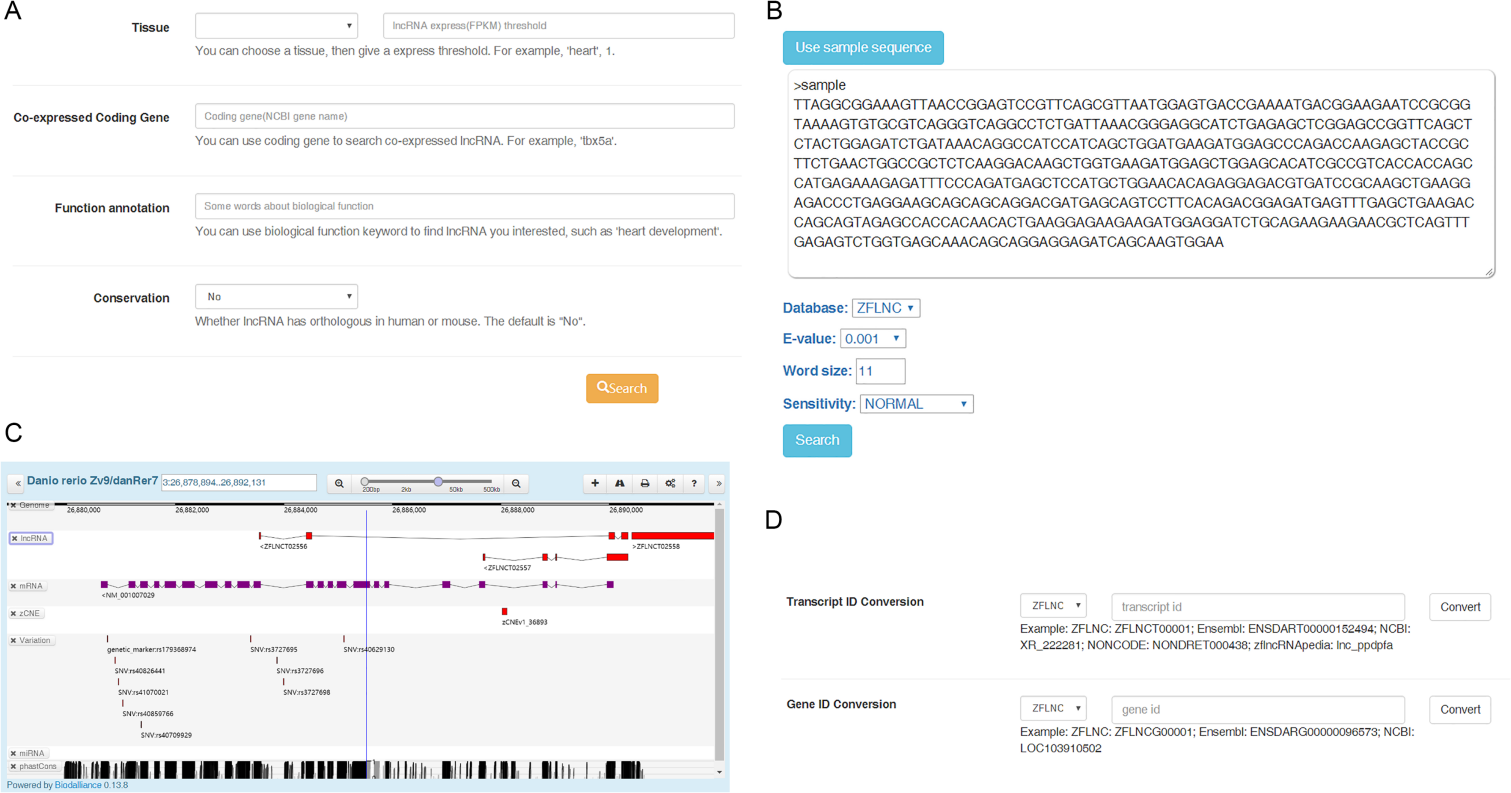
Usage of the database. **(A)** Filtering conserved functional lncRNA candidate through ‘Advanced functional lncRNA filtering’ function. **(B)** Finding zebrafish lncRNA through BLAST sequence similarity search. **(C)** Finding zebrafish lncRNA through sequence positions in GBrowser. **(D)** Converting IDs among diverse databases by using ‘ID Conversion’.

***Find conserved functional lncRNA candidate.*** The most common scenario for using ZFLNC is to find the interested lncRNA for further experimental study. In ‘Search’ module, you can use ‘Advanced functional lncRNA filtering’ function to find conserved functional lncRNAs based on four different parameters: ‘tissue’, ‘co-expressed coding gene’, ‘function annotation’ and ‘conservation’ (Figure 2A). For example, you can specify a condition that the expression (FPKM value) in heart is more than 1 and the ‘function annotation’ keyword is ‘heart’ to find some heart-related lncRNA candidates. Or you can find some conserved lncRNA candidates which are co-expressed with heart development-related gene tbx5a by setting ‘Co-expressed Coding Gene’ as ‘tbx5a’ and ‘Conservation’ as ‘Yes’.

#### 

***Convert other sources of lncRNA to ZFLNC.*** In ZFLNC, we offered many options to convert the lncRNA in other sources into ZFLNC. If you have a zebrafish lncRNA from existing database, and you want to know its annotation information in ZFLNC or another database, ‘ID Conversion’ module can convert ID among diverse databases (Figure 2D). For example, if you have a lncRNA ‘lnc_ppdpfa’ from zflncRNApedia, the ‘ID Conversion’ module can assist in the ID switch into ‘ENSDART00000152494’ in Ensembl or ‘ZFLNCT00001’ in ZFLNC. Another scenario is that you only know the sequence of one lncRNA, but now, you can use ‘BLAST’ module to find this lncRNA in ZFLNC through sequence similarity (Figure 2B). For example, using ‘BLAST’ module, you can find human MALAT1 homolog in zebrafish, ZFLNCT12716. At last, if you only know the genomic location of one lncRNA, you can use ‘Gbrowser’ module to search the lncRNA in ZFLNC (Figure 2C). For example, you can find lncRNA ZFLNCT15181, which is a highly conserved lncRNA (PhastCons: 0.94), through the genomic location danRer7 chr18: 265 594–269 844.

## Discussion and future developments

In this study, we constructed ZFLNC, a comprehensive database of zebrafish lncRNA. ZFLNC will provide an integrated platform with multiple resources for deep exploration of zebrafish lncRNA’s functions. More importantly, ZFLNC will facilitate to further interrogate mammalian lncRNA’s functions. By utilizing the lncRNA conservation annotation in ZFLNC, it is possible to migrate the handle of a lncRNA in human or mouse into its zebrafish conterpart. Moreover, we can exploit the GO/KEGG/OMIM function annotations and co-expression profiling of lncRNAs in ZFLNC to quickly lock the interested lncRNA as well as its function-related coding gene. Furthermore, a variety of powerful tools that had been established on zebrafish to test gene function, such as Morpholino knockdown and CRISPR/CAS9 genome editing technologies, will be bound to provide strong support for further studies on the functions of lncRNAs.

Comparing with protein-coding genes, lncRNAs are often less conserved in primary sequence (15, 40) and may express at lower levels and in a more tissue- and cell-specific manner (41). Therefore, it remains a big challenge to identify lncRNAs and elucidate its function. ZFLNC contains more than 10 000 zebrafish lncRNA genes from all kinds of sources whereas the number is far less than that of human lncRNA genes. In the future, we will continuously identify and collect more high-confidence lncRNAs and update ZFLNC at intervals.

Although large numbers of lncRNAs have been identified, the methods and resources of the systematic annotation of lncRNA are still limited (42). Using co-expression network to predict the function of lncRNA is widely accepted in the field, albeit with low accuracy (43). By referring to existing works in mammals (44–46), we have introduced a variety of conservation analysis methods to study the potential lncRNA orthology, hoping to provide new insights into finding functional lncRNAs and moreover, to help study their mammalian counterparts. In the future, we will continue to incorporate more types of data and analysis strategies to improve the lncRNA functional annotation.

In addition to collecting the most complete dataset and the most comprehensive annotations, in ZFLNC, we have established a user-friendly website and provided useful web-based tools to help the researchers to better access zebrafish lncRNA information. In the future, we will keep working on developing new tools to better visualize gene expression data and trends, in-depth explore zebrafish lncRNA-related functional gene network and more comprehensively inspect the diverse mechanisms of lncRNA function. We will update the website and fix bug regularly and update a major version of the website every 2 years. We promise to maintain ZFLNC database for 5 years or more.

## Notes

**Database URL**: http://www.zflnc.org
